# Polybrene Inhibits Human Mesenchymal Stem Cell Proliferation during Lentiviral Transduction

**DOI:** 10.1371/journal.pone.0023891

**Published:** 2011-08-26

**Authors:** Paul Lin, Diego Correa, Yuan Lin, Arnold I. Caplan

**Affiliations:** 1 Department of Biomedical Engineering, Case Western Reserve University, Cleveland, Ohio, United States of America; 2 Skeletal Research Center, Department of Biology, Case Western Reserve University, Cleveland, Ohio, United States of America; 3 Stanford Cancer Center, Stanford University, Stanford, California, United States of America; University of Medicine and Dentistry of New Jersey, United States of America

## Abstract

Human mesenchymal stem cells (hMSCs) can be engineered to express specific genes, either for their use in cell-based therapies or to track them in vivo over long periods of time. To obtain long-term expression of these genes, a lentivirus- or retrovirus-mediated cell transduction is often used. However, given that the efficiency with these viruses is typically low in primary cells, additives such as polybrene are always used for efficient viral transduction. Unfortunately, as presented here, exposure to polybrene alone at commonly used concentratons (1–8 µg/mL) negatively impacts hMSC proliferation in a dose-dependent manner as measured by CyQUANT, EdU incorporation, and cell cycle analysis. This inhibition of proliferation was observable in culture even 3 weeks after exposure. Culturing the cells in the presence of FGF-2, a potent mitogen, did not abrogate this negative effect of polybrene. In fact, the normally sharp increase in hMSC proliferation that occurs during the first days of exposure to FGF-2 was absent at 4 µg/mL or higher concentrations of polybrene. Similarly, the effect of stimulating cell proliferation under simulated hypoxic conditions was also decreased when cells were exposed to polybrene, though overall proliferation rates were higher. The negative influence of polybrene was, however, reduced when the cells were exposed to polybrene for a shorter period of time (6 hr vs 24 hr). Thus, careful evaluation should be done when using polybrene to aid in lentiviral transduction of human MSCs or other primary cells, especially when cell number is critical.

## Introduction

Mesenchymal stem cells (MSCs) were originally isolated from human bone marrow (BM) aspirates and characterized by their adherence to plastic and their ability to differentiate into multiple cell types, such as chondrocytes, adipocytes, and osteoblasts [1], [2]. Since then, the therapeutic use of MSCs has been expanded through genetic modification of the cell. For instance, MSCs have been found to increase cardiac myocyte survival in an acute myocardial infarction model when the cells were engineered to overexpress SDF-1 [3]. In another example, Kumar et al. showed that MSCs were able to increase their engraftment to BM, normally a very rare event [4], when α4 integrin was transiently upregulated [5]. MSCs can also be used to treat cancer by exploiting the naturally high tumor tropism of MSCs with cells genetically modified to secrete cancer-killing drugs and thus act as drug delivery vehicles. For instance, the infusion of human MSCs (hMSCs) transfected with an adenovirus to release IFN-β significantly increased animal survival in a mouse glioma model [6]. Additionally, the tumor tropism can be further enhanced by modifying hMSCs to express on their cell surface a single-chain antibody to the EGFRvIII found on glioblastoma multiforme cancer cells [7].

In the examples presented above, short-term expression of the engineered gene might be enough to generate a physiological response, and hence methods such as adenoviral transfection might be sufficient in those cases. However, long-term gene expression by MSCs is desirable for situations where sustained expression is required. For example, MSCs have been proposed as treatment for genetic disorders such as Osteogenesis Imperfecta, in which a mutation in the type I collagen gene causes severely weakened bone structure and high rates of bone fractures. In one potential gene-related therapy, the patient's MSCs would be harvested, the genetic mutation corrected *ex vivo*, and the engineered cells delivered to the patient [8]. Another important use of long-term gene expression is in cell tracking studies, particularly those that require either long-term monitoring of engraftment efficiency with a quantitative assessment (where stable expression over time is needed) or in situations where the cell population is expected to expand *in vivo*. In such cases, labeling of hMSCs with GFP [9], LacZ [10], or luciferase [11] has been used.

One method to obtain successful long-term expression is to transduce MSCs with a retrovirus or lentivirus. Unfortunately, incubating a retrovirus or lentivirus alone with hMSCs results in very low transduction efficiencies [9], and increasing the viral titers does little to improve the efficiency. It is often required to have an additive in solution, the most common being polybrene. Polybrene (hexadimethrine bromide) is a cationic polymer discovered to enhance retroviral transduction [12]. It is believed that it exerts its effects by neutralizing the negative electrostatic repulsion between the cell surface and the virus particles, allowing the latter to be adsorbed easily onto the cell surface [13]–[15]. This adsorption does not depend on the type of receptor and viral envelope and is also temperature-independent. Polybrene is considered non-toxic at low concentrations, but has been found to negatively affect cell proliferation in some cell types, such as keratinocytes, at concentrations greater than 10 µg/mL [16]. In our attempts to transduce hMSCs with lentivirus, we observed that hMSC proliferation was also affected by polybrene. However, the impact of polybrene on hMSCs has not been described previously.

We report here that polybrene alone can dramatically decrease hMSC proliferation, especially at the commonly used concentrations of 4 and 8 µg/mL. We found that after polybrene exposure hMSCs did not regain their proliferative capabilities even after 3 weeks in culture and that FGF-2, a potent mitogen for hMSCs [17], did not overcome the inhibitory effects of polybrene. These inhibitory effects were reduced by both a decrease in the time of exposure and in the dose of polybrene below 4 µg/mL. The inhibitory effects were also reduced by culturing the cells under simulated hypoxic conditions, which increased overall proliferation rates, although the negative effect of polybrene was still present.

## Results

### Effect of polybrene on proliferation

**Figure 1:**
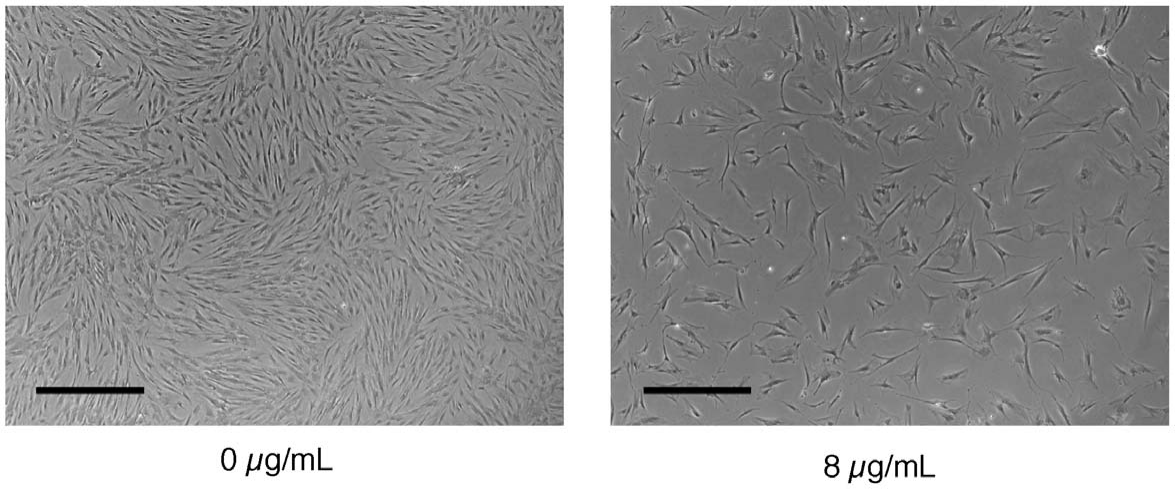
Phase contrast image of hMSCs exposed to polybrene. Phase contrast image of hMSCs either not exposed or exposed to 8 µg/mL of polybrene for 24 hr and then cultured for another 7 days in 100 mm dishes. Black bar  = 750 µm.

When hMSCs were cultured in the presence of 8 µg/mL of polybrene in 6-well plates, 100 mm dishes, or T175 flasks, their proliferation rate was dramatically decreased as observed by phase contrast microscopy (**Fig. 1**) and subsequent cell counts (data not shown). There was no difference in the initial attachment of the cells. This inhibition of proliferation was observed with multiple donors and with two different lots of hMSC-screened serum.

**Figure 2:**
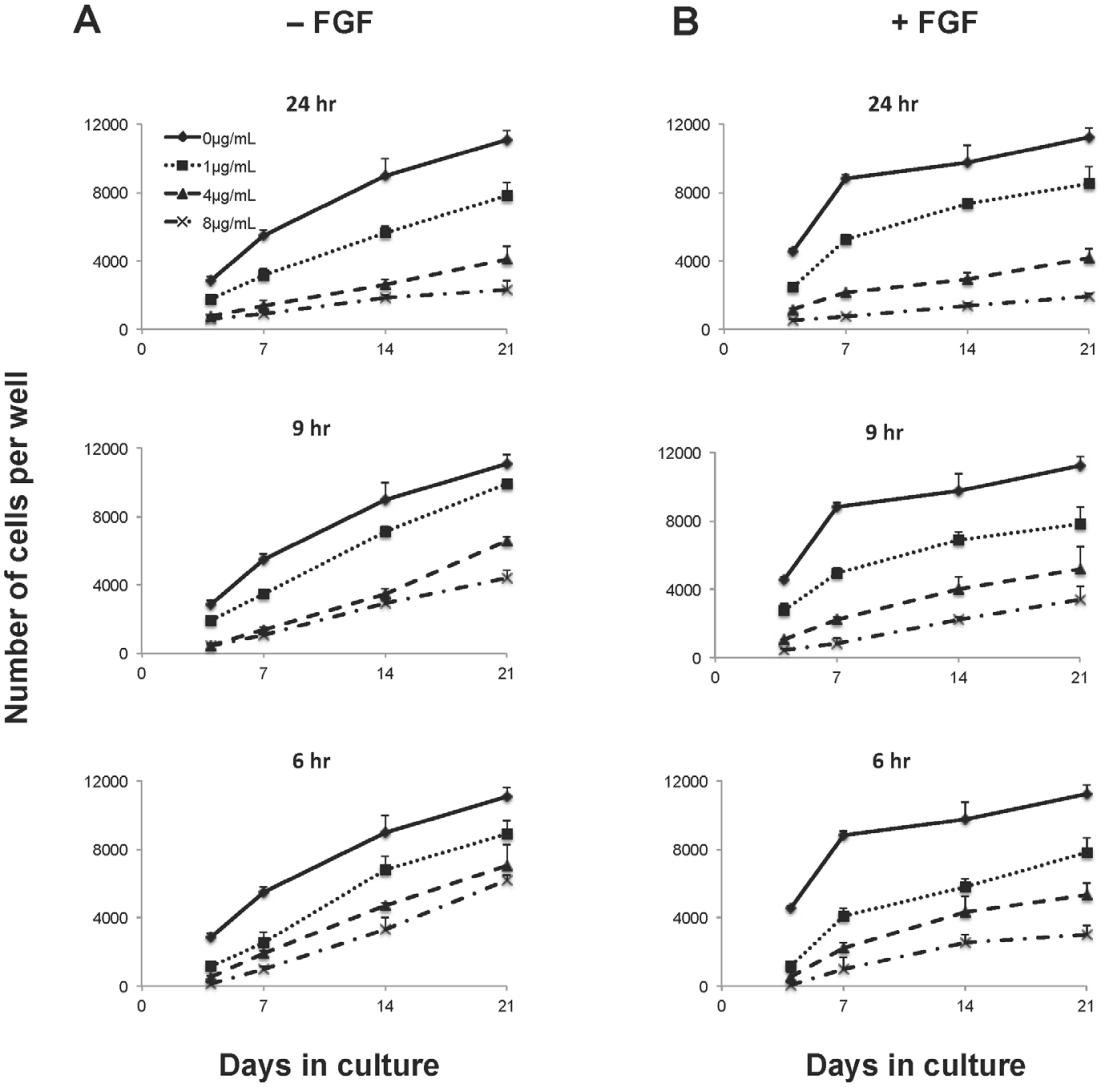
Inhibition of hMSC proliferation after polybrene exposure. Plot of cell number over 21 days as measured by the CyQUANT assay. Human MSCs were seeded at the same density in 96-well plates, exposed to different concentrations of polybrene for different lengths of time, and cultured in 50 µl of medium per well without (A) or with FGF-2 (B). The untreated controls are the same for the different time points. The graphs are all on the same scale. Values are mean ± SD.

To titrate the effect that different concentrations of polybrene have on hMSC proliferation and to determine whether exposure time has an impact, hMSCs were exposed to polybrene at concentrations ranging from 0 µg/mL to 8 µg/mL for different incubation times (**Fig. 2A**). Under standard culture conditions, the number of cells increased over 5-fold after 21 days. In contrast, with a polybrene treatment that is typically used for lentiviral transduction (8 µg/mL for 24 hr), the number of hMSCs only doubled within the same period of time (**Fig. 2A**). The difference in cell numbers between these groups can be observed as early as 7 days. Decreasing the concentration of polybrene reduced its negative effect on cell proliferation. However, even at 1 µg/mL of polybrene, the cell numbers never achieved a level equivalent to the control cells after 3 weeks of incubation. Decreasing the exposure time from 24 to 6 hr also reduced but did not eliminate the proliferative inhibition.

### Overcoming polybrene inhibition with FGF-2

In an attempt to overcome polybrene inhibition, cells were exposed to FGF-2, a known mitogenic agent. As expected, the presence of FGF-2 significantly increased the proliferation rate of the control cells (**Fig. 2B**). This effect was more dramatic during the first week of stimulation when a steep increase in cell number was followed by a plateau around the time of cell confluence. However, at higher concentrations of polybrene (4 and 8 µg/ml), the mitogenic effect was completely blocked and the proliferation rates were similar to those of polybrene treated cells in the absence of FGF-2 (**Fig. 2**). Phenotypically, while the hMSCs did not increase their proliferation, they did adopt a more spindle-like morphology typically found with FGF-2 treated control cells.

### Overcoming polybrene inhibition with hypoxia

**Figure 3:**
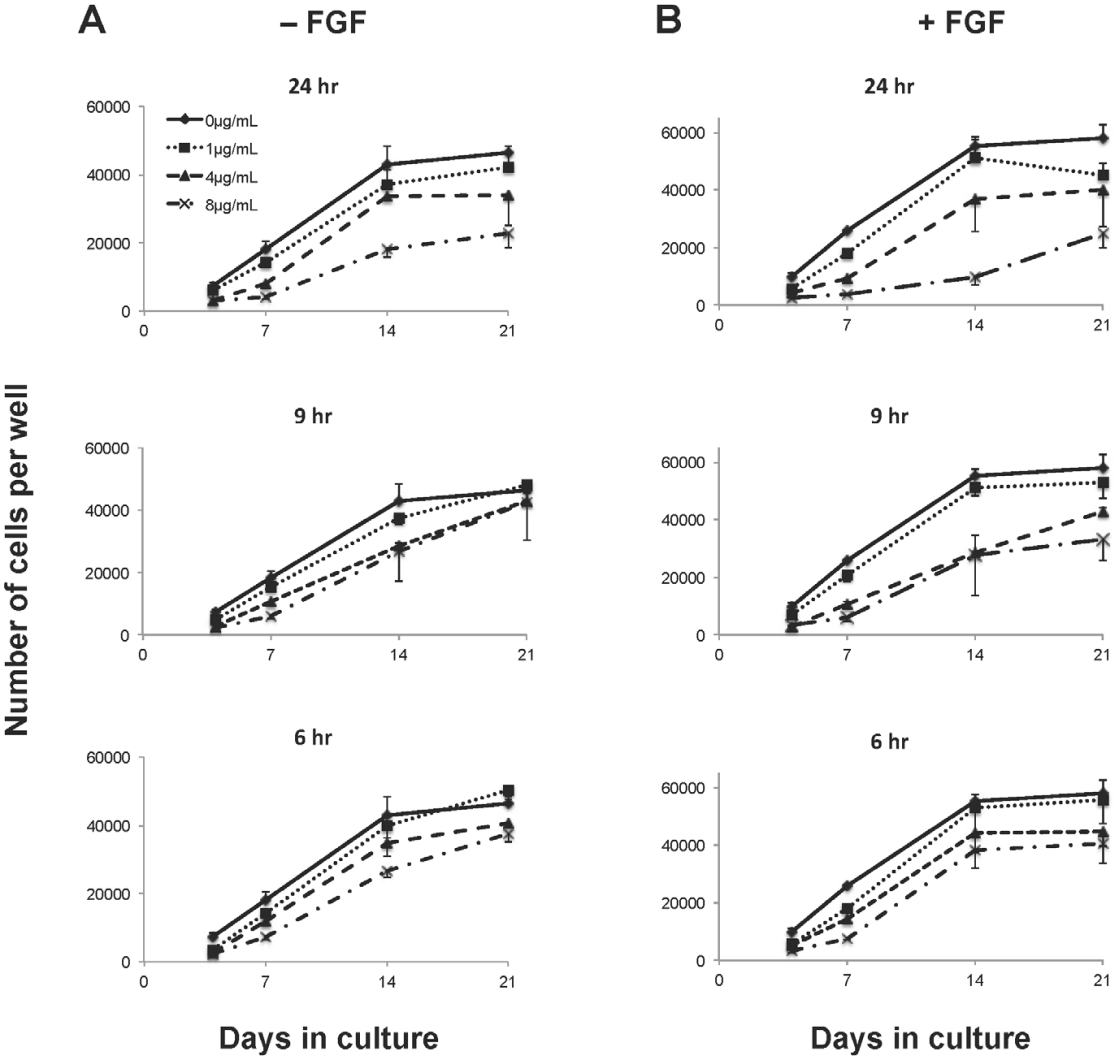
Effect of hypoxic conditions on polybrene inhibition of hMSC proliferation. Plot of cell number over 21 days as measured by the CyQUANT assay of hMSCs cultured in 200 µl of medium per well in the absence (A) or presence (B) of FGF-2. The untreated controls are the same for the different time points. The graphs are all on the same scale. Values are mean ± SD.

**Figure 4:**
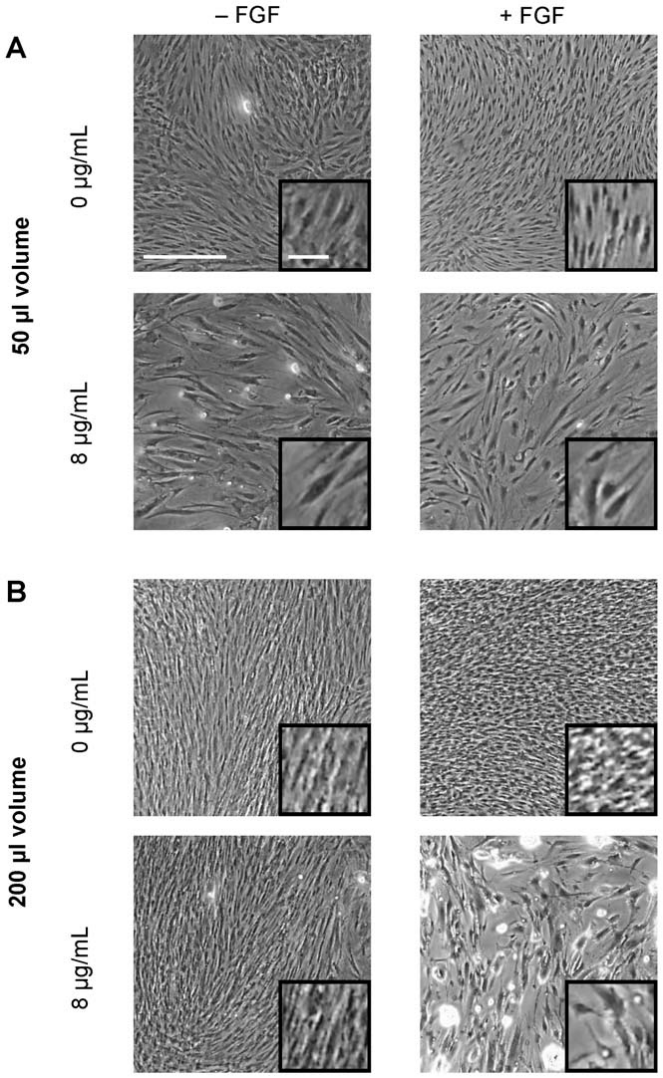
Phase contrast images of hMSCs cultured in 50 µl or 200 µl of medium per well. Phase contrast images of hMSCs from Fig. 2 and 3 that were either untreated or treated with to 8 µg/mL of polybrene for 24 hr. Cells were cultured for 14 days in either normal culture conditions (A) or under simulated hypoxic conditions (B). The medium was removed prior to image capture. All images are at the same magnification. The insert shows an increased magnification of the cells. White bar  = 500 µm (100 µm in insert).

To test whether hypoxic conditions can overcome the polybrene inhibition of hMSC proliferation, cells were cultured in a larger volume of medium to increase the medium height and thereby simulate hypoxic conditions. Human MSC cell numbers increased dramatically under these conditions (**Fig. 3**). Interestingly, the hypoxic conditions delayed the effects of contact inhibition as the control cells continued to proliferate rapidly up to day 14 (**Fig. 3** **)**, while there was a decrease in the proliferation rate under normal oxygen conditions after day 7 (**Fig. 2**). By phase contrast microscopy, a higher cell density was seen in cultures with 200 µl of medium compared to 50 µl of medium (**Fig. 4**). While the proliferation was still inhibited by polybrene in a dose-dependent way, hMSCs exposed to 8 µg/mL of polybrene in 200 µl of medium had a higher cell number per well at day 21 than untreated cells cultured in 50 µl of medium (23 k±4 k vs. 11 k±0.5 k). Overall the cell numbers at all polybrene concentrations were much closer to control under simulated hypoxic conditions.

### Cell cycle analysis

The number of cells obtained at a single time point is determined by the rate of cell proliferation and the rate of cell death. Therefore, at each time point trypan blue was used to measure dead cells to rule out cell death as a contributing factor in the decreased cell number. No difference was ever noticed between untreated and polybrene treated cultures with regard to the number of trypan blue stained cells (data not shown). However, interestingly, trypsinized cells exposed to polybrene were visibly larger in size when viewed under the microscope (data not shown).

**Figure 5:**
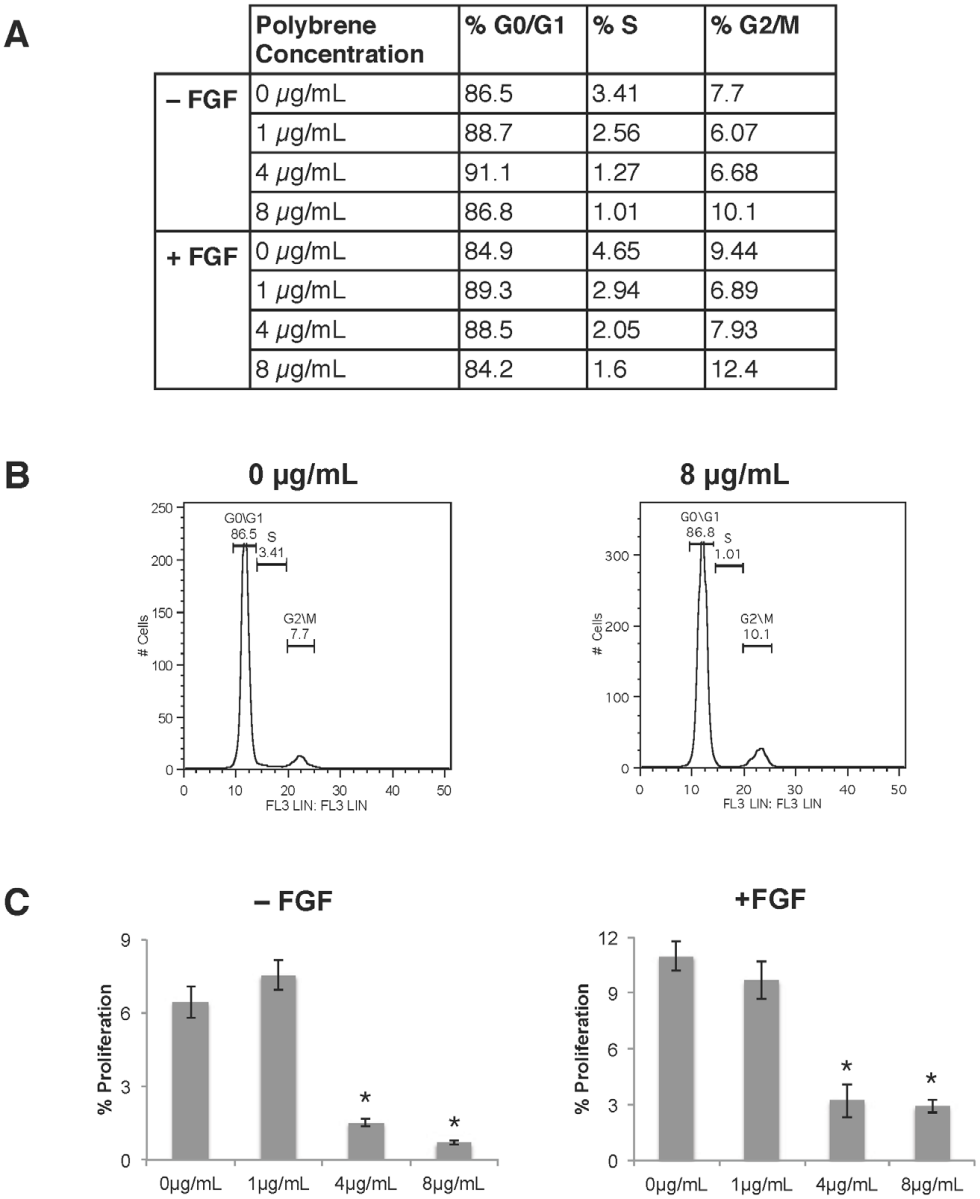
Cell cycle analysis with propidium iodide (PI) and EdU incorporation. Human MSCs were incubated with polybrene for 24 hr and then cultured for another 4 days in 100 mm dishes with 7 mL of medium per dish. (A) Cell cycle analysis with PI of hMSCs at different polybrene concentrations in the absence or presence of FGF-2. (B) Sample cell cycle histograms of hMSCs incubated in the absence of FGF-2 at 0 and 8 µg/mL of polybrene. (C) Proliferation measurement by EdU incorporation for hMSCs treated and untreated with FGF-2 at different polybrene concentrations (*p<0.001 vs. 0 µg/mL and 1 µg/mL, Tukey t-test). Values are mean ± SD.

To determine how polybrene affected the cell cycle, the cellular DNA was analyzed with propidium iodide (PI) and EdU (5-ethynyl-2′-deoxyuridine). Cell cycle analysis with PI indicated a decrease in the percentage of cells entering S phase with increasing concentrations of polybrene (**Fig. 5A-B**). As expected, there were higher numbers of cells in S phase in the presence of FGF-2, but the same decrease in cells entering S phase was still observed for cells treated with polybrene (**Fig. 5A**).

To measure the percentage of cells proliferating, hMSCs were incubated with EdU for 24 hr before harvest. Like BrdU, EdU is a thymidine analogue that will become incorporated into the DNA of dividing cells. This incorporation can then be detected by flow cytometry using the Click-iT EdU assay kit. As a function of polybrene concentration, there was a steep decrease between 1 µg/mL and 4 µg/mL for both FGF-2 treated and untreated cells (**Fig. 5C**).

### Effect of polybrene concentration on transduction efficiency

**Figure 6:**
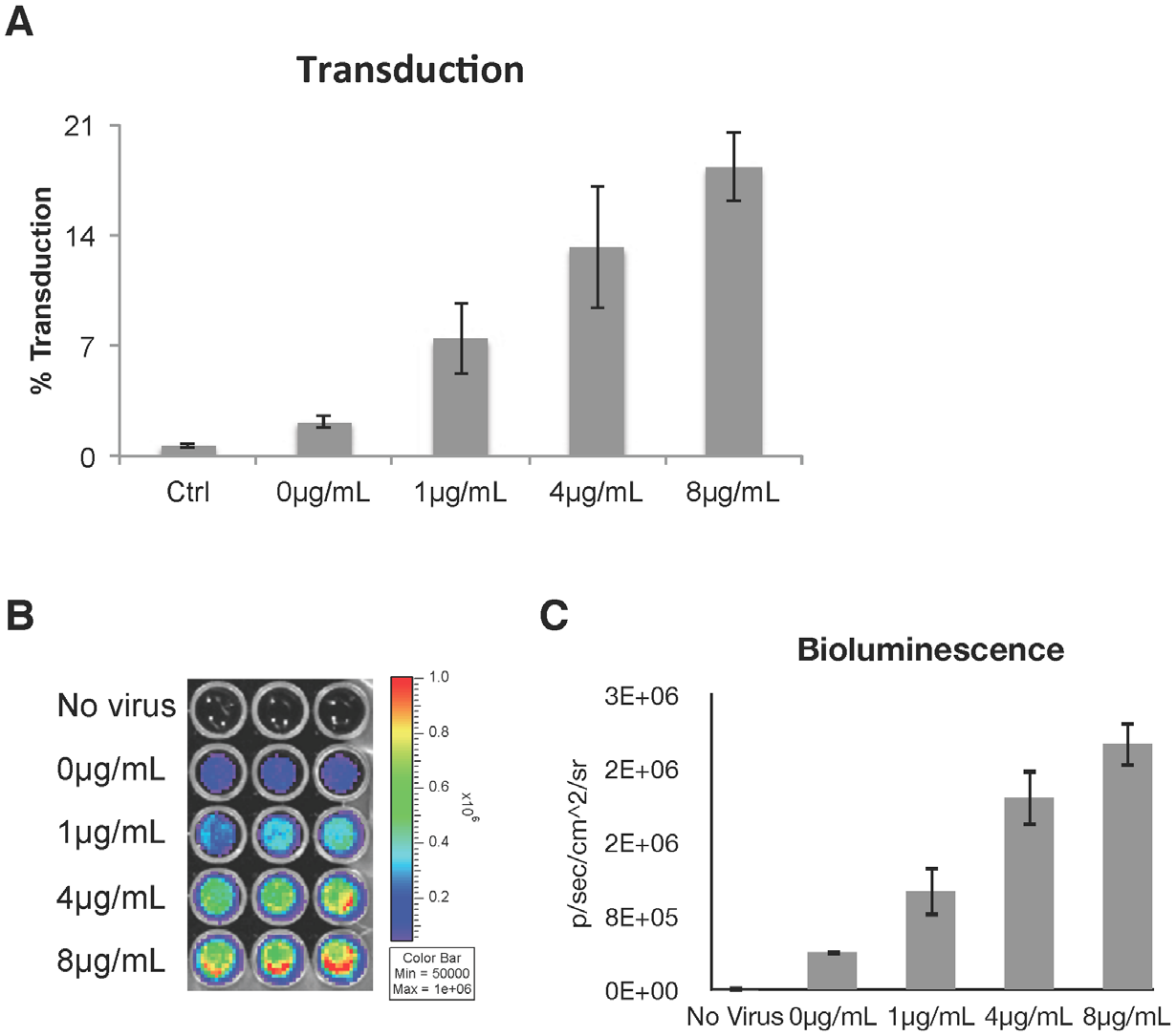
Lentiviral transduction of hMSCs with polybrene. Human MSCs were cultured with lentivirus containing a dual reporter gene that encodes a fusion protein of luciferase and mRFP. (A) Positive correlation of transduction efficiency with polybrene concentration as determined by mRFP flow analysis. (Spearman corr. coeff.  = 0.95, p<0.001) Values are mean ± SD. (B) Image of the bioluminescence signal from the transduced cells. (C) Chart of the number of photons detected in each well as a function of polybrene concentration. (Spearman corr. coeff.  = 0.97, p<0.001) Values are mean ± SD.

To measure if transduction can still be improved with low concentrations of polybrene, hMSCs were incubated with lentivirus containing a fused dual reporter gene that codes for firefly luciferase and monomeric red fluorescent protein (mRFP). The expression of mRFP, as measured by flow cytometry, showed a dose-dependent increase with increasing polybrene concentration (**Fig. 6A**). Transgene expression in the target cells was also closely correlated to the bioluminescence signal (**Fig. 6B & C**). The proliferation of transduced hMSCs was inhibited similar to hMSCs exposed to polybrene alone (data not shown).

## Discussion

From the experiments described in this paper, we found that culturing hMSCs in the presence of polybrene alone is sufficient to inhibit their proliferation under standard culture conditions. The CyQUANT assay showed that the number of cells in polybrene treated wells was lower than that for untreated wells. However, the assay gives the total number of cells in a well, and static cell numbers can only indicate either that the cells are dying and proliferating at a similar rate or that they are not proliferating. The fact that cell cycle analysis with PI showed fewer cells in S phase and that EdU incorporation over 24 hr was lower with increasing polybrene concentration provide evidence that the cells were not proliferating. Furthermore, there was no increase in trypan blue stained cells in polybrene treated cultures, which indicates that there was not an increase in cell death.

In an attempt to overcome the inhibition of proliferation by polybrene, hMSCs were incubated with FGF-2, which has been shown to be a potent mitogen for hMSCs [17]. Unlike cells not exposed to polybrene, for which FGF-2 had a clear mitogenic effect, there was no difference between FGF-2 treated or untreated cells at higher concentrations of polybrene. However, polybrene treated cells still adopted the spindle morphology typically found with FGF-2 treated hMSCs, suggesting that FGF-2 still had an effect on those cells.

An alternative method to increase hMSC proliferation is by culturing the cells under hypoxic conditions. Recent reports have indicated that hMSCs can increase their proliferation while maintaining their differentiation potential under low oxygen conditions [18]–[20]. We mimicked these conditions by increasing the height of the medium. According to Fick's first law of diffusion, the rate of oxygen delivery per unit area to the cell is inversely proportional to the height of the medium to the first order [21]. This equation has been confirmed experimentally [22], [23]. Our initial culture of hMSCs in 50 µl of medium per well of a 96-well plate had a medium height of approximately 156 µm, which is close to our standard medium height of approximately 127 µm in a 100 mm dish. We were able to decrease the amount of available oxygen by 25% by increasing the medium height four-fold with the use of 200 µl per well. After 3 weeks in culture, we obtained 4 times more cells with the increased medium height compared to the 50 µl medium height. We also report for the first time that FGF-2 and hypoxia have a synergistic effect in increasing hMSC proliferation, suggesting that the two act under different mechanisms. After 3 weeks, there were 26% more cells in the FGF-2 treated group. Also, it appears that the hypoxic condition was able to partially overcome contact inhibition. Based on the data in Fig. 2, confluence was reached at approximately 1.1×10^4^ cells per well. In contrast, growth plateaued at approximately 5×10^4^ cells per well under hypoxic conditions. This could be due to the fact that the cells were smaller as can be observed in Fig. 4. In addition, culturing under low oxygen did not completely antagonize the effect of polybrene as the cell yields were still lower compared to control. However, the effect of polybrene was greatly reduced at lower concentrations, and at high concentration (8 µg/mL) hypoxic conditions still resulted in twice as many cells as unexposed hMSCs cultured under non-hypoxic conditions. Collectively, these data support the beneficial effect of hypoxic culture conditions for hMSCs, even when polybrene is used as an adjuvant of cell transduction.

We also suspected that shortening the polybrene exposure time might mitigate the adverse effect of polybrene on hMSC proliferation. This did have a small effect under normal oxygen conditions, but the benefit was more noticeable with cells cultured under low oxygen. With a 6 hr exposure to 1 µg/mL of polybrene, the growth curve was almost identical to unexposed cells in either the presence or absence of FGF-2.

These studies emphasize the need to re-examine the appropriate conditions for viral transduction of hMSCs when polybrene is used. Using a lentivirus carrying a fused dual reporter gene, we found that there was a positive correlation between transduction efficiency and increasing concentrations of polybrene. Although 1 µg/mL of polybrene had the lowest percentage of transduced cells, there was still an over three-fold increase in transduction efficiency compared to transduction in the absence of polybrene. Furthermore, proliferation was inhibited by only 30% under standard culture conditions and 9% under hypoxic conditions. The lower transduction efficiency might not be problematic if the cells can proliferate to a greater degree so that it would be possible to obtain a larger number of transduced cells in the end. Hence, utilizing this lower dose of polybrene might be an acceptable compromise, especially when paired with low oxygen culture.

Polybrene is a widely used supplement in retroviral and lentiviral transduction. Unfortunately, the adverse effect of polybrene on the proliferation of hMSCs presents a technical problem. With a low proliferation rate, it becomes difficult to attain the large number of cells required for cellular therapies. Thus, the success of genetically modified hMSC treatments would initially require large quantities of virus and a large number of hMSCs, making pre-clinical experiments and potential therapy costly and difficult. Furthermore, viral transduction with polybrene could interfere with the engraftment and proliferation of transduced hMSCs *in vivo*. The fact that polybrene can affect hMSC proliferation and cell morphology also begs the question as to whether polybrene has other potential side effects on the cells. Thus, the use of polybrene with hMSCs must be done with caution.

## Materials and Methods

### Human MSC isolation and culture

Isolation of hMSCs was performed as described before [24], [25]. Briefly, bone marrow aspirates were obtained from the posterior superior iliac crest of healthy adult donors who gave written consent (IRB approved protocol from University Hospitals, Cleveland, OH). The cells were washed once with Dulbecco's Modified Eagle's Medium (DMEM-LG, Sigma Chemical) supplemented with 10% fetal bovine serum (FBS) that had been screened for hMSC culture [26]. The cells were centrifuged and then resuspended in 5 mL of medium and placed on top of 35 mL of 63% (v/v) Percoll gradient. After centrifuging at 460 g for 15 minutes, the top 25% of the gradient containing the nucleated cells was transferred to a new tube and washed again. The nucleated cells were then seeded at a density of 1.8×10^5^ per cm^2^ and cultured for 2 weeks before initial passage in DMEM-LG supplemented with 10% FBS. Medium was changed every 3-4 days for each passage. Cells were passaged every week with trypsin-EDTA (0.25% trypsin, 4 mM EDTA, Gibco) and seeded at 3–4.5×10^3^ per cm^2^ in 100 mm tissue culture dishes and cultured in 7 mL of medium. Passages 1 to 3 were used for the experiments described herein.

### Proliferation analysis

Trypsinized cells were washed and resuspended in medium at 2×10^4^ cells/mL with or without rhFGF-2 (final concentration  = 10 ng/mL, PeproTech) and with or without polybrene (Sigma Chemical) at a final polybrene concentration of 1, 4, or 8 µg/mL. The different conditions were seeded in 96-well plates at 1×10^3^ cells in 50 µl per well in triplicate and cultured at 37°C, 5% CO_2_. After 6, 9, or 24 hr, the medium was changed and subsequent medium changes occurred every 3–4 days with 50 µl of +/– FGF-2 medium (10 ng/mL). Plates were harvested on day 4, 7, 14, and 21 by removing the medium and placing the plates in the −80°C freezer until the day of analysis. The CyQUANT assay (Molecular Probes) was then performed on the wells following the manufacturer's instructions.

For hypoxic studies, the cells were resuspended at 5×10^3^ cells/mL and seeded in 96-well plates at 1×10^3^ cells in 200 µl per well in triplicate. The cells were cultured in 200 µl of medium per well for the duration of the experiment. The rest of the experiment was performed as described above for cells cultured in 50 µl of medium.

### Cell cycle analysis

#### Propidium iodide (PI)

Human MSCs were seeded with different concentrations of polybrene with or without FGF-2 (10 ng/mL) in 100 mm dishes. After 24 hr, the medium was changed with 7 mL of fresh medium, and the cultures were incubated for another 4 days. The cells were then trypsinized, and fixed in 70% ethanol at −20°C for 10 min. The cells were washed once with PBS, resuspended in RNase (0.2 mg/mL), and incubated at 37°C for 10 minutes. An equal volume of PI was added to obtain a final PI concentration of 50 µg/mL. The cells were then analyzed on an EPICS XL flow cytometer.

#### Click-iT EdU assay

Cells were seeded at 1×10^5^ per well in a 6-well plate in different concentrations of polybrene with or without FGF-2. After 24 hr, the medium was replaced with 1.5 mL of fresh medium and the cultures were maintained for another 4 days. EdU was added to the medium 24 hr before harvest to obtain a final EdU concentration of 10 µM. The cells were then trypsinized, and fixed in 4% formaldehyde. EdU incorporation was determined with the Click-iT EdU Alexa Fluor 647 flow cytometry assay kit (Molecular Probes) according to the manufacturer's instructions.

### Transduction with lentivirus

The reporter gene used is a modification of the triple fusion reporter vector described previously [27] (gift from Dr. Zhenghong Lee, Case Western Reserve University) that contains firefly luciferase (*fluc*), monomeric red fluorescent protein (*mrfp*), and herpes simplex thymidine kinase (*ttk*) domains in which the *ttk* domain was removed with BamHI. The gene is driven by the modified myeloproliferative sarcoma virus promoter (MND promoter). The second generation replication-incompetent lentivirus was generated using a three-plasmid system in 293T cells with the reporter plasmid, pCMVΔR8.91 (packaging vector), and pMD.G (vesicular stomatitis virus protein G pseudotyping vector). Viral titers were determined with 293T cells.

To transduce the hMSCs, 1×10^5^ cells per well were seeded in triplicate in 6-well plates with the lentivirus at a multiplicity of infection (MOI) of 5 and with a final polybrene concentration of 0, 1, 4, or 8 µg/mL. After 24 hr, the medium was replaced, and the cells were cultured for another 6 days with one additional medium change. The cells were trypsinized and analyzed on the iCyt Reflection flow cytometer for mRFP (ex: 561 nm, em: 615/30 Band Pass Filter) to determine the percentage of the cells that were transduced. The cells were also seeded onto black 96-well plates in triplicate at 5×10^3^ cells per 150 µl of medium and 25 µl of luciferin (Luciferase Assay System, Cat# E1500, Promega) per well. The plate was imaged for 3 min with a field of view of 13 cm using the Xenogen IVIS Imaging 200 Series system (Caliper Life Sciences, Hopkinton, MA). Grids were drawn around each well and the number of photons was calculated using the Living Image software v2.5 (Caliper Life Sciences, Hopkinton, MA).
